# Predictors of effective kangaroo mother care, exclusive breastfeeding, and skin-to-skin contact among low birthweight newborns in Amhara, Ethiopia

**DOI:** 10.7189/jogh.14.04114

**Published:** 2024-09-09

**Authors:** Moses Collins Ekwueme, Abel Zemedkun Girma, Abebe Gebremariam Gobezayehu, Melissa F Young, John N Cranmer

**Affiliations:** 1 Nutrition and Health Sciences Program, Laney Graduate School, Emory University, Atlanta, Georgia, USA; 2 Hubert Department of Global Health, Emory University, Atlanta, Georgia, USA; 3 Center for the Study of Human Health, Emory University, Atlanta, Georgia, USA; 4 Brown School, Washington University in St. Louis, St. Louis, Missouri, USA; 5 Emory University, Atlanta, Georgia, USA; 6 Bahir Dar University, Bahir Dar, Ethiopia; 7 Emory-Ethiopia, Addis Ababa, Ethiopia

## Abstract

**Background:**

Globally, 20% of all newborns are born with low birth weight (LBW). There is, therefore, an urgent need to expedite the delivery of high-impact, evidenced-based, and low-cost interventions such as kangaroo mother care (KMC (defined as continuous skin-to-skin care) and exclusive breastfeeding for this vulnerable group.

**Methods:**

A multinational World Health Organization (WHO)-supported consortium created and tested the impact of locally-specific and globally-informed phases of KMC care on KMC uptake/scale-up across multiple sites. Here we report on the study of KMC predictors that is nested within Amhara’s KMC implementation trial in Amhara, Ethiopia. We used multivariate logistic regression phases to identify diverse predictors of KMC, skin-to-skin contact, and exclusive breastfeeding at hospital discharge and day 28 of life.

**Results:**

We analysed data from 860 LBW newborns. At day 28, implementation period (adjusted odds ratio (aOR) = 3.2–5.0), hospital facility (aOR = 3.0–4.6), and having multiple births (aOR = 0.31) were the strongest predictors of effective KMC. Meanwhile, previous death of a newborn, type of health facility where delivery occurred, and previous LBW delivery were predictors of effective KMC at both time points. No single factor predicted KMC, skin-to-skin contact, and exclusive breastfeeding at all time points and across all implementation periods. Having multiple births was a negative predictor for skin-to-skin contact, while the implementation period and having older fathers (>29 years) were strong positive predictors for exclusive breastfeeding at both discharge and day 28. Mothers with a previous history of neonatal death and current skin-to-skin-care uptake strongly predicted exclusive breastfeeding uptake at both time points. At discharge, however, having a history of preterm birth and neonatal death strongly predicted exclusive breastfeeding uptake, while multiple current births, current very LBW newborns, and the use of standard binders decreased the likelihood of exclusive breastfeeding.

**Conclusions:**

To achieve the effective KMC coverage target of ≥80% in Ethiopia, KMC scale-up phases may have to consider the key predictors of KMC, EBF, and SSC to effectively target beneficiaries.

An estimated 20 million newborns are born with low birth weight (LBW) each year, 95% of which are born in low- and middle-income countries (LMICs) [1]. LBW infants born prematurely or small for gestational age are at a higher risk of neonatal morbidity and mortality and are more susceptible to infections/sepsis, neurocognitive developmental delays, and chronic non-communicable diseases later in life [2-5]. Globally, LBW newborns account for 70% of all newborn deaths [6]. Consequently, neonatal mortality remains disproportionately higher in LMICs, particularly in sub-Saharan Africa, with a neonatal mortality rate of 28 deaths per 1000 live births in 2019 [7]. In the past two decades, Ethiopia recorded notable reductions in infant and under-five mortality, similar to those observed globally. However, the country’s newborn mortality ratio only decreased by 15% over 14 years [8]. Due to this lack of improvement in newborn survival, the government set a goal of lowering newborn mortality to 21 per 1000 live births by 2025 [9].

Kangaroo mother care (KMC) is a cost-effective intervention for reducing neonatal mortality rates, reducing sepsis, and improving other health outcomes for LBW infants [10,11]. It was first introduced in Colombia in 1978 as an alternative to incubators. Recent work by the World Health Organization (WHO) and global collaborating centres defined effective KMC as ≥8 hours of skin-to-skin contact (SSC) between newborn/caregiver per 24 hours and exclusive breastfeeding (EBF)/breast milk provision (e.g. expressed breast milk) [12,13]. Despite the expanding body of research supporting KMC, global uptake remains low, with implementation restricted to certain clinical settings such as referral centres in capital cities [14]. Even though KMC was introduced to Ethiopia as early as 1996 and the Ministry of Health set a goal in line with the Global Every Newborn Action Plan target of reaching 80% of LBW infants by 2025 [15], coverage in Ethiopia has historically remained limited, with only <5 to 10% of eligible newborns receiving any type of KMC [16]. Therefore, it is important to understand the facilitators and barriers of effective KMC and its two components to maximise uptake of KMC-based interventions [17,18].

KMC brings numerous health benefits through SSC and EBF. For example, the former plays a major role in regulating the newborn's body temperature by providing it with warmth. Relatedly, hypothermia was associated with 69% of deaths of LBW newborns treated in neonatal intensive care units in Ethiopia [19]. Moreover, incubators are not commonly available in health facilities in LMICs, so alternative methods for maintaining body temperature are widely adopted, such as polyethylene plastic wraps, warm delivery rooms, or newborn hats and socks. Yet a large systematic review has shown that, through SSC, KMC reduced the risk of hypothermia by 72% compared to conventional care and provided other benefits such as improved mother-infant bonding [20]. Furthermore, despite abundant evidence that human breast milk is the optimal food for newborns [21], particularly for LBW neonates, several challenges to EBF uptake among LBW infants have been well documented [10,22-24].

Implementation science methods are increasingly being used to create, test, and scale KMC in the sub-Saharan Africa and Southeast Asia regions, with the end goal of increasing population-based effective KMC coverage. These methods primarily use a participatory action approach and in-depth interviews to gather information from key health care workers and community stakeholders, with several modifications incorporated into the KMC scale-up phase at three different phases (phase 0, 1, and 2) [16-18]. Nonetheless, the effectiveness of these KMC scale-up initiatives has yet to be fully evaluated, with evidence from Oromia, Amhara, the Tigray regions of Ethiopia, and Southern Nations showing that KMC coverage in the 24 hours before discharge and seven-days post-discharge ranges from 54–82% and 38–60%, respectively [13,16,25,26]. While these scale-up phases incorporated several factors identified as constraints to SSC and EBF uptake [16], KMC coverage still trails behind the global coverage target of ≥80% by 2025. Accordingly, there remain critical LBW-specific gaps in KMC uptake, and further research is needed to identify and address barriers and facilitators of KMC and both of its two components – EBF and SSC – across the community-to-facility continuum, at discharge through 28 days of life (D28). These efforts could also help maximise the uptake of KMC and optimise future cycles of KMC scale-up and phase development/refinement. Hence, we designed this study to quantify the barriers and facilitators of KMC, EBF, and SSC at discharge and D28 using mother-LBW newborn pairs from five KMC-implementing health facilities in the Amhara Region of Ethiopia (**Box 1**, **Box 2**).

Box 1 **Key findings.**

- The strongest overall predictor of KMC, EBF and SSC is the implementation phase period. This was achieved through the continuous refinement of KMC scale-up strategies, addressing context-specific barriers and facilitators of effective KMC identified throughout the study.
- The uptake of KMC and its two components (EBF and SSC) is variable and depends on multiple factors including patient, obstetric, social, and implementation factors. For example, mothers with multiple current births have a strongly decreased likelihood of practising any of the three interventions.

Box 2 **Key implication**

- To achieve high population coverage of effective KMC in Ethiopia, future KMC scale-up strategies may benefit from using the specific barriers (e.g. multiple births) and facilitators (e.g. hospital type, time since implementation) identified here to provide more targeted support for mothers who are less likely to adopt the intervention. These strategies may include intensified coaching/support during hospital admission or after discharge.
- These Ethiopia-specific findings may enable hospital clinicians to intensify support for mothers/families who are less likely to practice SSC or EBF – particularly during hospitalisation – to increase the sustained adoption of KMC through the newborn period.

## METHODS

### Population, setting, and sample

Emory-Ethiopia was a member of a multinational WHO-supported consortium that created and tested the impact of locally-specific and globally-informed phases of KMC care on KMC uptake in Ethiopia and India. It led the KMC implementation trial in Amhara, Ethiopia [13], within which this study of KMC predictors is nested [27].

We designed this study to identify the factors that predict effective KMC, along with sufficient skin-to-skin contact and EBF at both hospital discharge and D28 of life. For this purpose, we identified variables from the existing KMC data set that may influence the uptake of KMC, SSC, and EBF/breast milk feeding, as defined by the WHO KMC Study Group [13,16,27]. This data set contained clinical, obstetric, sociodemographic, and intervention variables for the entire KMC study period (from April 2017 to April 2019) at three different time points: discharge, day seven, and D28 of life. The data was collected across three different phase implementation periods: phase 0 (April and October 2017), phase 1 (November 2017 to May 2018), and phase 2 (June 2018 to April 2019) [13]. A detailed description of the differences in research and programme activities across implementation periods has been detailed elsewhere [13]. For our analysis, all data in this nested study were deidentified following a formal data usage agreement. We used sociodemographic data from enrolment (at birth) and KMC, SSC, and EBF outcome data at hospital discharge and D28. Since D28 data were primarily collected once the newborns had been discharged at home for some time, we used this time point as a proxy for sustained SSC, EBF, and KMC practices. Data were collected on mobile tablets using REDCap [28] and exported to Stata, version 16.0 (StatCorp LLC, College Station, Texas, USA) for cleaning and analysis.

There were four categories of predictors in the data set:

1. Socioeconomic factors: household income, geographic residence, and household size;
2. Obstetric factors: mother’s history of pregnancies and childbirths;
3. Newborn clinical factors; birthweight, delivery type, and gestational age;
4. Intervention-related factors: those factors that might influence outcomes such as the use of KMC binders or newborn diapers.

### Statistical analysis

We used a multivariate logistic regression model to predict KMC, SSC, and EBF at two time points – hospital discharge and D28. We constructed this final model using four iterative steps [29]:

1. Describing, transforming, and categorising outcome and predictor variables;
2. Performing bivariate analysis to explore relationships between individual predictors and each of the three clinical outcomes (KMC, EBF, SSC) at two time points using univariate logistic regression (ULR);
3. Constructing a multivariate logistic regression model (MLR) for each of the outcomes (KMC, SSC, EBF) using both theory/previous literature and empiric univariate relationships between predictor and outcome;
4. Selecting the final MLR model by balancing the highest possible C-statistic (also known as the area under the receiver operator characteristics curve (AUC)) to produce final MLR models that had both variables with high effect sizes (adjusted odds ratio (aOR) and statistical significance at *P* < 0.05.

### Descriptive analysis

We used conventional numerical and graphical descriptive analysis to investigate the variables based on their level of measurement and distribution. For example, we visualised continuous variables using histograms and box plots and described them using medians and interquartile ranges (IQRs), as none were normally distributed. This allowed us to identify extreme/unanticipated values or missing data prior to our analysis. Next, we examined the relationships between predictor and outcome variables using both graphic (e.g. stratified box plots or histograms for continuous variables) and numeric techniques (e.g. medians and IQRs). Otherwise, we reported frequencies and percentages for categorical variables.

We then tested for differences between variables using univariate statistics. For example, we compared continuous variables across three time periods using the Kruskal-Wallis H test. For categorical variables, we tested differences using Pearson χ^2^ test of independence (or Fischer’s exact test if any category had six or fewer observations). Finally, we descriptively examined relationships between predictor and outcome variables using graphical (e.g. stratified box plots) and numeric techniques (e.g. odds ratios (ORs), H test, or χ^2^/Fisher exact test). This helped us understand the possible relationships between the potential predictors and the outcome of interest.

### Predictor variables

As part of the data cleaning, variable description, and empirical bivariate relationships between predictor and outcomes, we recategorised several variables for intuitive interpretation, statistical significance, and highest predictive power (e.g. treating continuous birthweight or household income in empirical categories). For example, we collapsed some variables like the number of previous births into two categories (singletons and multiple births). For others like the number of previous LBW newborns for each mother, we had to strategically determine the cut-off points for transforming these continuous variables into category-based empirical distribution. To create these cut-off points, we examined empirical distributions and identified where groups based on new cut-off point categories had statistically and clinically significant differences in the outcome based on the cut-off points themselves. For example, we converted continuous household income into quartiles and parental age, birthweight category, obstetric history, and household size into categories.

### Logistic regression models

After empirical or theoretical recategorisation of select variables, we set up ULR models to quantify the strength and direction of the relationship between the predictors and each of the three outcomes (KMC, EBF, SSC) at two time points (discharge and D28). Variables with high effect sizes, ORs above or below 1, and statistically significant *P*-values (at *P* < 0.05) were considered for the final multivariate logistic regression model for each outcome/period.

At this stage, we also eliminated potentially collinear variables in the MLR models. We eliminated predictor variables from a model if they had high non-parametric correlation coefficients (e.g. Spearman’s Rho) or strong theoretical relationships. In instances where we detected collinearity, we tested out the variable that should be retained and removed based on the effect sizes of the variables and/or C-statistic of the overall model. Next, we examined the potential MLR models to create models with the largest effect size (very high or low OR) and significant *P*-values. The goal of the final model was to retain only clinically and statistically significant variables, while obtaining the highest possible AUC.

We began with all possible predictor variables from the four categories and made step-wise deletions of variables that had low effect size or were not statistically significant. In our initial draft MLR model for each outcome, we reintroduced variables one-by-one to the MLR that have high effect sizes from ULR models to determine if they should be added back into the draft MLR. We tested the impact of these additions on the model’s overall AUC. We retained any reintroduced variables that retained high effect sizes and statistical significance while increasing the C-statistic. Our final MLR models for each outcome/time period contained predictors with the highest effect size/clinical significance and the highest C-statistic. Finally, after refining the models, we confirmed there was no collinearity using the variance inflation factor (VIF) using the pre-defined criterion of ten.

## RESULTS

### Infant characteristics

We included 860 LBW newborns (<2000 g) in this study based on WHO KMC Study Group weight cut-offs [27]; 16.3% were born during implementation phase 0, 23.7% during phase 1, and 59.9% during phase 2. The overall median birthweight across implementation phases was 1670 g (IQR = 1500–1800) (**Table 1**); it did not differ between models (*P* = 0.137) (**Table 1**). The median gestational age of 33 weeks did not differ by period (*P* = 0.757), nor did the 20% incidence of very premature births at 25–31 weeks (*P* = 0.724) or the 20.7% overall incidence of very LBW (VLBW) (<1500 g; *P* = 0.183). Moreover, 31.3% of births were multiples with subtle differences by phase, with a range from 27–39% (*P* = 0.001). The mode of birth did not differ by phase; 84.8% of newborns were delivered vaginally, 11.3% by Caesarean section, and 4.0% were forceps-assisted (*P* = 0.103).

**Table 1:** Infant, family and facility characteristics by phase period*

Predictors	Total	Phase 0	Phase 1	Phase 2	*P*-value
**Clinical factors**					
Participants	860 (100)	140 (16.3)	23.7 (204)	59.9 (515)	
Birthweight group					0.183†
*VLBW (1000–1500 g)*	178 (20.7)	33 (23.6)	49 (24.0)	96 (18.6)	
*LBW (1500–2000 g)*	682 (79.3)	107 (76.4)	155 (76.0)	419 (81.4)	
Gestational age category					0.724‡
*Very premature (25–31 weeks)*	174 (20.5)	24 (17.3)	38 (19.0)	111 (21.9)	
*Premature (32–37 weeks)*	629 (74.2)	109 (78.4)	151 (75.5)	369 (72.6)	
*Normal (38–42 weeks)*	45 (5.31)	6 (4.32)	11 (5.50)	28 (5.51)	
Current number of births – singletons	591 (68.7)	88 (62.9)	124 (60.8)	378 (73.4)	0.001†
Delivery type					0.103‡
*Normal vaginal*	729 (84.8)	122 (87.1)	170 (83.3)	436 (84.7)	
*Assisted vaginal, forceps*	34 (4.0)	2 (1.43)	5 (2.45)	27 (5.24)	
*Caesarean section*	97 (11.3)	16 (11.4)	29 (14.2)	52 (10.1)	
Health facility					0.004§
*Primary 1*	103 (12.0)	17 (12.1)	28 (13.8)	58 (11.3)	
*Primary 2*	61 (7.10)	5 (3.57)	6 (2.96)	50 (9.71)	
*Referral hospital*	345 (40.2)	57 (40.7)	75 (37.0)	213 (41.4)	
*General 1*	82 (9.55)	8 (5.71)	20 (9.85)	53 (10.3)	
*General 2*	268 (31.2)	53 (37.9)	74 (36.5)	141 (27.4)	
≥4 ANC visits	323 (40.1)	40 (30.5)	65 (34.6)	217 (44.7)	0.003†
**Obstetric history**					
No abortions	750 (87.4)	123 (87.9)	165 (80.9)	461 (89.9)	0.005†
≥1 preterm	791 (92.1)	135 (96.4)	188 (92.2)	467 (90.9)	0.085‡
LBW category					0.001†
*≤1 LBW infant*	626 (72.9)	94 (67.6)	133 (65.2)	398 (77.3)	
*>1 LBW infants*	233 (27.1)	45 (32.4)	71 (34.8)	117 (22.7)	
Stillbirth category – none (0)	821 (95.6)	135 (97.1)	188 (92.2)	497 (96.5)	0.036‡
≥1 neonatal deaths	75 (8.7)	16 (11.4)	23 (11.3)	36 (7.0)	0.084†
Birth spacing category					0.192†
*≤3 y (10–36 months)*	168 (33.2)	24 (32.0)	39 (34.5)	105 (33.0)	
*>3–5 y (37–72 months)*	244 (48.2)	35 (46.7)	46 (40.7)	163 (51.3)	
*>5 y (73–288 months)*	94 (18.6)	16 (21.3)	28 (24.8)	50 (15.7)	
**Socioeconomic factors**					
Residence					0.769†
*Rural*	535 (62.4)	84 (60.0)	129 (63.9)	321 (62.3)	
*Urban*	323 (37.7)	56 (40.0)	73 (36.1)	194 (37.7)	
Residential zone					0.006†
*South Gondar*	417 (48.5)	69 (49.3)	106 (52.2)	241 (46.8)	
*West Gojjam*	402 (46.8)	58 (41.4)	85 (41.9)	259 (50.3)	
*Other*	40 (4.7)	13 (9.3)	12 (5.9)	15 (2.9)	
Household size	3–5 (4)	3–5 (3)	3–5 (4)	3–5 (4)	0.0228‖
Phone ownership					0.335†
*Mother owns phone*	509 (61.2)	76 (56.3)	28 (64.3)	305 (61.4)	
*Mother does not own phone*	323 (38.8)	59 (43.7)	71 (35.7)	192 (38.6)	
Annual household income – ETB 10 000, MD (IQR)	2.82 (2.00–4.00)	2.20 (1.50–4.40)	2.50 (1.90–4.00)	3.00 (2.30–3.90)	0.0001‖
Income quartiles (annual)					<0.001†
*1st and 2nd quartile (ETB≤28 200)*	430 (50.0)	92 (65.7)	115 (56.4)	222 (43.1)	
*3rd and 4th quartile (ETB>28 200)*	50.0 (430)	48 (34.3)	89 (43.6)	293 (56.9)	
**Parental factors**					
Mother age in years (16–45), MD (IQR)	27.0 (24.0–30.0)	27.0 (23.5–30.0)	26.5 (23.0–32.0)	27.0 (24.0–30.0)	0.623‖
Mother age category in years					0.744†
*≤24*	251 (29.2)	42 (30.0)	63 (30.9)	145 (28.2)	
*>24*	609 (70.8)	98 (70.0)	141 (69.1)	370 (71.8)	
Father age in years (20–53), MD (IQR)	32.0 (29.0–38.0)	30.0 (27.5–35.0)	33.0 (28.0–40.0)	33.0 (29.0–38.0)	0.0038‖
Father age category in years					0.057†
*≤29*	136 (29.4)	38 (38.0)	32 (30.5)	65 (25.3)	
*>29*	327 (70.6)	62 (62.0)	73 (69.5)	192 (74.7)	
Parental age gap in years, MD (IQR)	6 (3–8)	4 (2–7)	5 (3–9)	6 (4–8)	0.0009‖
Parental age gap category in years					<0.001†
*≤3*	120 (25.9)	39 (39.0)	33 (31.4)	48 (18.7)	
*>3*	343 (74.1)	61 (61.0)	72 (68.6)	209 (81.3)	
Mother is literate	395 (45.9)	77 (55.0)	108 (52.9)	209 (40.6)	0.001†
Father is literate	451 (52.9)	92 (66.7)	121 (59.6)	238 (46.6)	0.000†
Father’s living situation – living with mother	796 (92.6)	130 (92.9)	188 (92.2)	477 (92.6)	0.966†
Mother’s occupation category					0.075†
*Working mother*¶	520 (60.6)	82 (58.6)	111 (54.7)	327 (63.6)	
*Housewife*	337 (39.4)	58 (41.4)	92 (45.3)	187 (36.4)	
Father’s occupation category					0.113†
*Farmer*	523 (61.1)	82 (58.6)	114 (55.9)	327 (63.9)	
*Non-farmer*	333 (38.9)	58 (41.4)	90 (44.1)	185 (36.1)	
**Implementation factors at discharge**					
Binder use					<0.001†
*KMC standard binder*	553 (71.6)	7 (6.36)	117 (60.6)	429 (91.7)	
*Traditional binder*	219 (28.4)	103 (93.6)	76 (39.4)	39 (8.33)	
Newborn diaper use					0.025†
*Did not use*	381 (44.5)	59 (42.5)	106 (52.7)	215 (41.8)	
*Did use*	475 (55.5)	80 (57.6)	95 (47.3)	300 (58.3)	
At D28 binder use					0.033‡
*Traditional binder*	445 (89.5)	45 (83.3)	107 (87.0)	293 (91.9)	
*KMC standard binder*	32 (6.4)	3 (5.6)	10 (8.1)	19 (6.0)	
*No binder*	20 (4.0)	6 (11.1)	6 (4.9)	7 (2.2)	
Infant diaper use					0.037†
*Did not use*	310 (63.0)	26 (47.3)	79 (65.3)	205 (64.9)	
*Did use*	182 (34.0)	29 (52.7)	42 (34.7)	111 (35.1)	

ANC – antenatal care, D28 – day 28, KMC – Kangaroo Mother Care, LBW – low birth weight

*Presented as n (%) unless specified otherwise.

†Pearson χ^2^ test of independence.

‡Fischer’s exact test.

§Should be Fischer’s exact test, but Stata produces error.

‖Kruskal-Wallis H-test.

¶Working mother includes: farmer, skilled labour, unskilled labour, professional, merchant, petty trade, and other occupations.

### Obstetric, family, and social characteristics

The median maternal age of 27 years was consistent across phases (IQR = 24–30; *P* = 0.623) (**Table 1**). Fathers were older than mothers, with a median age of 32 years (IQR = 29–38). The median age difference between male and female partners was 6 years (IQR = 3–8), with 74.1% of partners having more than a three-year age gap. Literacy rates among fathers and mothers were 52.9% and 45.9%, respectively. Further, 92.6% of fathers were living with the newborn’s mother, about 61% were farmers, and 14.2% were professionals. Meanwhile, 60.6% of mothers worked and 39.4% were housewives. Newborns were from families with a median household size of four persons (IQR = 3–5), which varied slightly by phase period (*P* = 0.0228) (**Table 1**). The median household income was ETB 28 200 (IQR = 20 000–40 000). It increased from 22 000 to 30 000 across the three phase periods (*P* = 0.0001). Lastly, 81.6% of families owned their homes and 61.2% of mothers owned mobile phones.

During pregnancy, 40.1% of women received ≥4 antenatal care visits; this varied from 31–45% across the phases (*P* = 0.006). Among mothers, 87.4% had no previous abortions, 92.1% had a previous preterm birth, 27.1% had previously birthed a LBW newborn, 4.4% had one or more stillbirths and 8.7% had one or more previous neonatal deaths (**Table 1**). The 48-month median time since the previous pregnancy (IQR = 36–72) was consistent across phase periods (*P* = 0.7123).

### Implementation and facility characteristics

Newborns were primarily from Specialty Hospital 1 (40.2%) and General Hospital 1 (31.2%); there were some phase/period-based recruitment differences across the five facilities which represents the step-wise implementation phase that progressively added facilities (*P* = 0.004) (**Table 1**). Moreover, 62.4% of newborns were from rural households. Participants were nearly equally from South Gondar (48.5%) and West Gojjam (46.8%) with some subtle phase/period-based differences (*P* = 0.006).

### Key outcomes of KMC, SSC, and EBF

Effective KMC is composed of two sub-indicators – SSC (defined as ≥8 hours per day) plus EBF/exclusive breast milk feeding. At hospital discharge, effective KMC increased by 15.8% from 59.0% in phase 0 to 74.8% in phase 2 (**Table 2**).

**Table 2:** Primary outcomes by phase period and birthweight category*

	SSC†		EBF‡		Effective KMC (SSC and EBF)	
	**Discharge**	**D28**	**Discharge**	**D28**	**Discharge**	**D28**
**Total**	701 (81.5)	402 (46.7)	636 (75.5)	382 (74.8)	594 (70.6)	343 (67.1)
**Phase period**						
Phase 0	85 (60.7)	27 (19.3)	114 (82.0)	41 (67.2)	82 (59.0)	24 (39.3)
Phase 1	164 (80.4)	99 (48.5)	142 (71.4)	88 (72.1)	135 (67.8)	80 (65.6)
Phase 2	451 (87.6)	276 (53.6)	379 (75.4)	252 (77.1)	376 (74.8)	239 (73.1)
**Birthweight category**						
Very LBW§	160 (19.7)	63 (16.1)	119 (18.7)	59 (15.5)	111 (18.7)	56 (16.3)
LBW‖	651 (80.3)	328 (83.9)	517 (81.3)	323 (84.6)	483 (81.3)	287 (83.7)

D28 – day 28, EBF – exclusive breastfeeding, KMC – kangaroo mother care, LBW – low birth weight, SSC – skin-to-skin contact

*Presented as n (%) unless specified otherwise.

†Defined as ≥8 h of skin-to-skin contact per day.

‡Defined as neonate only fed breastmilk.

§Defined as 1000–1500 g.

‖Defined as 1500–2000 g.

More substantively, effective KMC increased by 33.2% at D28 of life from 39.3% in phase 0 to 73.1% in phase 2. SSC at discharge increased by 26.9% from 60.7% in phase 0 to 87.6% in phase 2. At D28, SSC increased by 34.3% across the same periods (19.3% to 53.6%). Changes in EBF rates were more variable. EBF increased most across the three phase periods at D28 time point; it rose by 9.9% from 67.2% in phase 0 to 77.1% in phase 2. Unlike KMC and SSC, EBF was highest at discharge during phase 0 at 82.0% and then declined by 10.6% during phase 1 (to 71.4%), after which it increased slightly during phase 2 compared to phase 1 (to 74.5%). Although all implementation phases had a high effect size EBF, the proportion of people EBF declined compared to the baseline in phase 1.

### Univariate prediction

We used univariate logistic regression to predict all three outcomes – effective KMC, EBF, and SSC. We predicted these three outcomes at hospital discharge and the end of the neonatal periods (D28).

Across both time points (discharge, D28), several variables statistically predicted lower uptake of all three outcomes – KMC, EBF, and SSC (*P* < 0.05). Having multiple births substantively decreased KMC (OR = 0.26), EBF (OR = 0.22), and SSC (OR = 0.13) at discharge, as well as at day 28 (KMC: OR = 0.34, EBF: OR = 0.30, SSC: OR = 0.51) (**Table 3**). Similarly, the number of previous preterm births statistically decreased the odds of KMC (OR = 0.55), EBF (OR = 0.54), and SSC (OR = 0.38) at discharge and at day 28 (KMC: OR = 0.52, EBF: OR = 0.63, SSC: OR = 0.64). Having ≥2 previous preterm births was also detrimental to KMC, EBF, and SSC (**Table 3**). Having one or more previous LBW newborns had a similar negative impact on KMC at discharge (OR = 0.24) and D28 (OR = 0.30), as well as EBF and SSC at both time points (**Table 3**).

**Table 3:** Univariate odds of effective KMC, skin-to-skin contact and exclusive breastfeeding (at discharge and 28 d of life)*

	SSC		EBF		Effective KMC (SSC and EBF)	
**Predictor by category**	**Discharge**	**D28**	**Discharge**	**D28**	**Discharge**	**D28**
**Clinical factors**						
Very LBW category (ref: LBW<2000 g)	0.70 (0.08)	0.68 (0.03)	0.65 (0.02)	0.60 (0.05)	0.69 (0.04)	0.80 (0.35)
Multiple current births (ref: singletons)	0.13 (<0.01)	0.51 (<0.01)	0.22 (<0.01)	0.30 (<0.01)	0.26 (<0.01)	0.34 (<0.01)
Number of ANC visits	0.92 (0.30)	0.98 (0.72)	0.83 (0.01)	0.75 (<0.01)	0.84 (0.02)	0.83 (0.03)
*≥4 ANC visits (ref: <4 ANC visits)*	0.79 (0.19)	0.84 (0.22)	0.58 (<0.01)	0.47 (<0.01)	0.63 (<0.01)	0.53 (<0.01)
Delivery type (ref: normal vaginal)						
*Assisted (forceps)*	1.00 (0.99)	0.69 (0.30)	0.32 (<0.01)	0.21 (<0.01)	0.39 (0.01)	0.24 (0.01)
*Caesarean section*	0.65 (0.10)	0.92 (0.72)	0.62 (0.04)	0.71 (0.26)	0.79 (0.32)	0.72 (0.25)
*Assisted and C-sections (ref: normal vaginal)*	0.72 (0.16)	0.86 (0.42)	0.51 (<0.01)	0.53 (0.02)	0.65 (0.03)	0.56 (0.02)
Birthing facility (ref: referral hospital)						
*Primary 1*	1.38 (0.28)	3.61 (<0.01)	3.63 (<0.01)	4.02 (<0.01)	3.68 (<0.01)	2.99 (<0.01)
*Primary 2*	1.81 (0.14)	2.24 (<0.01)	3.63 (<0.01)	5.30 (<0.01)	3.23 (<0.01)	4.35 (<0.01)
*General 1*	1.22 (0.53)	1.72 (0.03)	2.21 (0.01)	2.04 (0.04)	2.42 (<0.01)	2.04 (0.03)
*General 2*	1.35 (0.15)	1.17 (0.35)	4.63 (<0.01)	3.36 (<0.01)	3.71 (<0.01)	3.45 (<0.01)
Gestational age in weeks	1.06 (0.10)	1.00 (0.96)	1.06 (0.04)	1.02 (0.65)	1.07 (0.02)	1.02 (0.57)
Gestational age (ref: premature (32–37 weeks)						
*Very premature (25–31 weeks)*	0.85 (0.44)	0.79 (0.18)	0.69 (0.05)	0.54 (0.01)	0.73 (0.08)	0.64 (0.05)
*Normal (38–42 weeks)*	1.20 (0.67)	0.78 (0.43)	0.89 (0.74)	0.44 (0.05)	0.93 (0.83)	0.42 (0.03)
**Obstetric history**						
≥1 abortion (ref: no abortion)	1.00 (0.997)	1.16 (0.47)	0.93 (0.76)	1.35 (0.37)	0.86 (0.49)	1.51 (0.18)
Number of preterm births	0.38 (<0.01)	0.64 (<0.01)	0.54 (<0.01)	0.63 (<0.01)	0.55 (<0.01)	0.52 (<0.01)
*No preterm births (ref: ≥1 preterm)*	1.07 (0.85)	1.31 (0.29)	0.66 (0.13)	0.75 (0.40)	0.80 (0.42)	0.82 (0.54)
>2 preterm births *(ref: ≤1)*	0.17 (<0.01)	0.45 (<0.01)	0.22 (<0.01)	0.31 (<0.01)	0.28 (<0.01)	0.32 (<0.01)
*>1 LBW birth (ref: ≤1)*	0.13 (<0.01)	0.48 (<0.01)	0.20 (<0.01)	0.27 (<0.01)	0.24 (<0.01)	0.30 (<0.01)
*≥1 stillbirths (ref: no stillbirths)*	0.62 (0.21)	0.73 (0.36)	0.93 (0.86)	0.57 (0.24)	0.91 (0.80)	0.83 (0.70)
*≥1 neonatal death (ref: no deaths)*	1.35 (0.37)	0.83 (0.45)	2.12 (0.03)	1.18 (0.68)	1.65 (0.10)	1.02 (0.96)
Birth space category (ref: 37–72 months)						
*10–36 months*	1.45 (0.16)	0.87 (0.48)	1.46 (0.10)	1.52 (0.17)	1.38 (0.15)	1.35 (0.28)
*73–288 months*	1.46 (0.24)	1.09 (0.73)	1.50 (0.16)	1.73 (0.13)	1.49 (0.15)	1.35 (0.35)
**Socioeconomic factors**						
Urban residence (ref: rural residence)	0.93 (0.70)	0.94 (0.64)	0.74 (0.07)	0.99 (0.97)	0.75 (0.06)	1.03 (0.86)
Residential zone (ref: South Gondar)						
*West Gojjam*	1.08 (0.69)	1.32 (0.05)†	0.64 (0.01)	0.92 (0.69)	0.66 (0.01)	0.83 (0.33)
*Other*	0.33 (0.01)†	0.36 (0.01)	1.00 (0.99)	0.97 (0.97)	0.38 (<0.01)	0.61 (0.41)
Household size	0.91 (0.07)	1.05 (0.24)	1.01 (0.86)	1.09 (0.17)	1.07 (0.16)	1.07 (0.21)
Mother does not own phone (ref: mother owns phone)	0.79 (0.19)	0.85 (0.25)	0.79 (0.16)	0.59 (0.01)	0.77 (0.10)	0.66 (0.04)
Household income (ETB 10 000)	0.97 (0.07)	0.97 (0.08)	0.98 (0.16)	0.95 (0.04)	0.98 (0.14)	0.96 (0.10)
*High income, quartiles 3–4, ETB>28 200 (ref: ETB≤28 200)*	1.02 (0.93)	1.12 (0.41)	0.83 (0.25)	0.97 (0.90)	0.91 (0.52)	1.08 (0.69)
**Parental factors**						
Mother’s age						
*Mother’s age in years (16–45)*	1.00 (0.93)	1.01 (0.53)	0.99 (0.46)	1.01 (0.69)	1.00 (0.87)	1.00 (0.86)
*Younger, ≤24 years (ref: >24 years)*	1.18 (0.40)	1.09 (0.58)	1.39 (0.07)	1.03 (0.89)	1.17 (0.37)	1.14 (0.54)
Father’s age						
*Father’s age in years (20–53)*	0.99 (0.64)	1.00 (0.87)	0.97 (0.03)	0.97 (0.12)	0.98 (0.24)	0.98 (0.23)
*Younger, ≤29 years (ref: >29 years)*	0.78 (0.30)	0.85 (0.42)	1.40 (0.16)	1.75 (0.07)	1.08 (0.74)	1.30 (0.36)
Age gap						
*Parental age gap in years*	0.97 (0.34)	0.96 (0.16)	0.94 (0.02)	0.92 (0.03)	0.95 (0.07)	0.95 (0.17)
*≤3 years (ref: >3 years)*	0.91 (0.72)	0.77 (0.23)	1.60 (0.07)	1.63 (0.13)	1.55 (0.06)	1.06 (0.84)
Literate mother (ref: illiterate mother)	0.83 (0.29)	0.99 (0.93)	0.98 (0.89)	0.99 (0.97)	0.84 (0.26)	1.14 (0.50)
Illiterate father (ref: literate father)	1.26 (0.19)	1.40 (0.01)	0.82 (0.23)	0.89 (0.56)	1.01 (0.97)	0.95 (0.80)
Father not living with mother (ref: father living with mother)	0.66 (0.17)	1.01 (0.98)	0.71 (0.24)	1.01 (0.97)	0.74 (0.28)	0.71 (0.32)
Mother’s occupation – housewife (ref: working mother)	0.93 (0.67)	1.32 (0.05)	0.90 (0.51)	0.92 (0.67)	0.94 (0.67)	0.83 (0.32)
Father’s occupation – non-farming father (ref: farmer)	0.96 (0.83)	0.90 (0.45)	0.90 (0.50)	1.02 (0.93)	0.85 (0.30)	1.15 (0.47)
**Implementation factors**						
Phase period (ref: phase 0)						
*Phase 1*	2.65 (<0.01)	3.95 (<0.01)	0.55 (0.03)	1.26 (0.49)	1.47 (0.10)	2.94 (<0.01)
*Phase 2*	4.56 (<0.01)	4.83 (<0.01)	0.67 (0.10)	1.64 (0.10)	2.06 (<0.01)	4.19 (<0.01)
**Implementation factors at discharge**						
Standard binder use (ref: traditional binder)	15.9 (<0.01)	2.07 (0.01)	0.62 (0.02)	1.13 (0.61)	1.14 (0.46)	1.73 (0.01)
Infant used diapers (ref: infant did not use diapers)	1.90 (0.04)	0.68 (0.09)	0.35 (<0.01)	0.41 (<0.01)	0.42 (<0.01)	0.45 (<0.01)
**Implementation factors at D28**						
Binder use (ref: traditional binder)						
*No binder*		0.33 (0.03)		0.21 (0.01)		0.17 (<0.01)
*Standard binder*		0.88 (0.74)		0.37(0.04)		0.48 (0.11)
Newborn used diapers (ref: did not use diapers)		0.60 (0.01)		0.51 (0.01)		0.49 (<0.01)

ANC – antenatal care, D28 – day 28, EBF – exclusive breastfeeding, KMC – kangaroo mother care, LBW – low birth weight, ref – reference, SSC – skin-to-skin contact

*Presented as odds ratio (*P*-value) unless specified otherwise. *P*-values <0.05 are considered statistically significant.

Compared to the high-volume specialty hospital, all other facilities had statistically significant increased odds of effective KMC at discharge (range of ORs = 2.4–3.7; *P*-values <0.001) and at D28 (range of ORs = 2.0–4.5; range of *P*-values = 0.00–0.03). There were similar odds of EBF and SSC at three facilities for SSC on D28 of life. Although the health facility had a large effect size on EBF at discharge, it was not statistically significant (**Table 3**). All implementation phase periods had a high effect size for increased KMC and SSC and the phase period was statistically significant for predicting seven of eight indicators (KMC and SSC at discharge and D28 for both phase periods). There was a dose-response increase in these indicators, suggesting that KMC and SSC improved substantively with each sequential implementation phase. The odds of effective KMC at D28 increased from 1 to 2.9 and 4.2 across the three phases (all statistically significant at *P* < 0.001). Although the odds of EBF sequentially increased at D28 across phase periods, it was only marginally significant for phase 2 (phase 1 OR = 1.3, *P* = 0.49; phase 2 OR = 1.6, *P* = 0.10). In contrast to all other indicators (KMC, EBF, SSC) and time points, the odds of EBF decreases at discharge compared to the baseline phase period (phase 1 OR = 0.55, *P* = 0.03; phase 2 OR = 0.65, *P* = 0.10).

Several predictors impacted only one or two of the three indicators. For example, women with assisted births through caesarean sections or forceps were about half as likely to provide EBF and KMC at discharge and D28 (range of ORs = 0.51–0.65; range of *P*-values = 0.00–0.02) (**Table 3**). The odds of SSC also decreased among those with assisted deliveries (range of ORs = 0.72–0.86), but this trend was not statistically significant (range of *P*-values = 0.16–0.42). In turn, completing 4 or more antenatal care visits was associated with decreased odds of KMC and EBF (range of ORs = 0.47–0.63; *P*-values <0.001). Very LBW delivery decreased the odds of all three indicators (KMC, EBF, SSC). These decreases were significant for four of the six phase periods/outcome combinations. The odds of EBF decreased compared to phase 0 at both additional time points (discharge: OR = 0.65, *P* = 0.02; D28: OR = 0.62, *P* = 0.05). This was similar to VLBW’s impact on KMC at discharge (OR = 0.69; *P* < 0.001) and SSC at D28 (OR = 0.68; *P* = 0.03). Although very LBW decreased the odds of EBF at discharge and KMC at D28, the findings were not statistically significant (**Table 3**). Interestingly, some factors had differential impacts on KMC’s two components – SSC and EBF. For example, the use of insert diapers and binders.

### Multivariate prediction and phase performance

The MLR predictors for outcomes (KMC, SSC, EBF) and time periods (discharge, D28) varied (**Table 4**). Our post-hoc VIF test for co-linearity did not indicate any phases contained co-linear predictors (mean VIF = 2.7–5.6) (**Table 4**).

**Table 4:** Multivariate predictors of KMC-related outcomes (KMC, EBF, SSC) at discharge and D28 of life*

	Effective KMC (EBF and SSC)		EBF		Sufficient SSC	
**Predictors**	**Discharge**	**D28**	**Discharge**	**D28**	**Discharge**	**D28**
**Obstetric factors**						
						
Current birth – multiple births (ref: singleton)	0.43 (<0.01)	0.31 (<0.01)		0.42 (0.01)	0.13 (<0.01)	0.19 (<0.01)
Very LBW, <1500 g (ref: ≥1500 g)				0.45 (0.05)		
Obstetric history						
*Previous LBW infant (ref: ≤1 LBW infant)*	0.38 (<0.01)		0.38 (<0.01)			
*≥1 preterm birth (ref: no previous preterm births)*			2.35 (0.02)			
Previous neonatal deaths (ref: no neonatal deaths)	2.97 (<0.01)		2.87 (<0.01)			
**Social factors**						
Household size (number of persons, 1–11)	1.14 (0.002)					
Older father in years, >29 years (ref: ≤29 years)					2.22 (0.03)	
**Implementation factors**						
Health facility (ref: referral hospital)						
*Primary 1*	3.82 (<0.01)	3.75 (<0.01)	5.87 (<0.01)	4.37 (<0.01)		
*Primary 2*	2.92 (<0.01)	3.86 (<0.01)	4.65 (<0.01)	7.48 (0.01)		
*General 1*	2.55 (<0.01)	2.97 (<0.01)	3.51 (<0.01)	2.22 (0.12)		
*General 2*	4.25 (<0.01)	4.56 (<0.01)	6.88 (<0.01)	2.89 (0.01)		
Phase period (ref: 0)						
*Phase 1*	1.50 (0.12)	3.17 (<0.01)	0.12 (<0.01)		14.1 (<0.01)	10.4 (<0.01)
*Phase 2*	2.25 (<0.01)	5.02 (<0.01)	0.13 (<0.01)		29.2 (<0.01)	10.5 (<0.01)
Binder use (ref: Traditional binder)						
No binder				0.45 (0.30)		
KMC standard binder				0.35 (0.05)		
Effective SSC (ref: <8 hours/day)			26.9 (<0.01)	6.98 (<0.01)		
EBF (ref: not EBF)					37.2 (<0.01)	10.7 (<0.01)
Phase performance, AUC	78.0	75.8	87.1	83.4	91.8	85.8
Phase, mean VIF	3.8	3.5	3.5	2.7	4.5	5.6

AUC – area under the curve, D28 – day 28, EBF – exclusive breastfeeding, KMC – kangaroo mother care, LBW – low birth weight, ref – reference, SSC – skin-to-skin contact, VIF – variance inflation factor

*Presented as adjusted odds ratio (*P*-value) unless specified otherwise.

#### KMC

Six variables predicated effective KMC at discharge from the hospital. The greatest increases in KMC were driven by hospital facility (range of aORs = 2.6–4.3; *P* < 0.001) (**Table 4**) and previous newborn death (aOR = 3.0; *P* < 0.001). Further, effective KMC increased across both phase periods, and significantly so in the final model (aOR = 2.3; *P* < 0.001). Two factors predicted decreased KMC adoption at discharge – having a previous LBW newborn (aOR = 0.4; *P* < 0.001) and having current multiple births (aOR = 0.43; *P* < 0.001). This multivariate logistic regression model had a solid predictive performance, with an AUC of 78.0%.

Three of the variables predicting KMC at discharge in the MLR model also predicted KMC at D28 – health facility, phase period, and current multiple births. The effect size for the implementation phase period increased at D28 compared to KMC at discharge, but was similar for the other variables (phase period, multiple births). The aOR for effective KMC was 3.2 (*P* < 0.001) during phase 1 and 5.0 (*P* < 0.001) during phase 2. The aOR by facility ranged from 3.0 to 4.6 (*P* < 0.001). Having multiple births was equally negative to KMC at D28 (aOR = 0.31; *P* < 0.001). This simpler model predicting KMC at D28 compared to discharge retained solid model performance, with an AUC of 75.8%.

### SSC

Four variables predicted SSC at discharge from the hospital; one was protective against SSC. Having multiple births greatly decreased the adjusted odds of SSC to 0.13 (*P* < 0.001). As the implementation period increased, the adjusted odds of SSC greatly increased. The aOR of SSC was 14.1 in phase 1 (*P* < 0.001), and it more than doubled during phase 2 (aOR = 29.2; *P* < 0.001). Newborns with older fathers (>29 years compared to ≤29) were more likely to receive SSC (aOR = 2.2; *P* = 0.03). EBF greatly increased the likelihood of practicing SSC (aOR = 37.2; *P* < 0.001). These four variables powerfully predicted SSC, with a high AUC of 91.8%.

Three of the four variables predicting SSC at discharge also predicted it at D28; paternal age did not predict sustained SSC. At D28, multiple births reduced the likelihood of SSC as it did at discharge (aOR = 0.19, *P* < 0.001). SSC increased dramatically during implementation phase periods 1–2 compared to phase 0. The effect size in both periods were similar (phase 1 aOR = 10.4, *P* < 0.001; phase 2 aOR = 10.5, *P* < 0.001). Concurrently practicing SSC increased the likelihood of EBF nearly 11-fold (aOR = 10.7; *P* < 0.001). The overall model performance for SSC at D28 was powerful with an AUC of 85.8%.

#### EBF

The variables predicting EBF at discharge and D28 varied. Only SSC and health facility were predictive of EBF at both time points. At discharge, three variables predicted increased EBF and three predicted decreased EBF. Having a previous LBW decreased the adjusted odds of EBF to 0.38 (*P* < 0.001), while having previous preterm births increased the odds of EBF (aOR = 2.35; *P* < 0.001). In contrast to KMC and SSC at both time points, the odds of EBF at discharge decreased in both phases (phase 1: aOR = 0.12, *P* < 0.001; phase 2: aOR = 0.13, *P* < 0.001), which is consistent with the decreasing raw counts of EBF in phases 1 and 2 compared to phase 0. As with KMC, having a previous neonatal death nearly tripled the aOR of EBF (aOR = 2.9; *P* < 0.001). The aOR of EBF was much higher at four facilities with three and half- to nearly 7-fold increases (aOR = 3.5–6.9, *P* < 0.001). Receiving SSC significantly increased the aOR for breastfeeding to 26.9 at discharge (*P* < 0.001). This multivariate model for EBF at discharge was robust with an AUC of 87.1%.

Five variables predicted EBF at D28; only two of the five were predictive at discharge. Two variables increased the likelihood of EBF – receiving SSC (aOR = 7.0; *P* < 0.001) and receiving care at three of the four hospitals (range of ORs = 2.9–7.5; range of *P*-values = <0.001–0.01). One hospital had a high effect size but it was not significant (aOR = 2.2; *P* = 0.12). Three variables predicted at least 55% decreased likelihood of EBF – multiple current births (aOR = 0.42; *P* = 0.01), very LBW of current newborn (<1500 g; aOR = 0.45; *P* = 0.05), and the use of standard binders to support the KMC position compared to traditional binders (aOR = 0.35; *P* = 0.05). This model of EBF at D28 was robust with an overall AUC of 83.4%.

## DISCUSSION

### Summary of findings

We designed this study to assess the predictors of effective KMC and its two main components – EBF and SSC – both at discharge and D28. We used a sample of 860 LBW newborn-mother dyads from five KMC-implementing hospitals in the Amhara region of Ethiopia. At both time points, effective KMC was predicted by the implementation phase period, a previous newborn death, health facility, multiple current births, and a previous LBW delivery. For SSC, having multiple births was a negative predictor while EBF, implementation phases, and having older fathers (>32 years) were strong positive predictors at both discharge and D28. In predicting EBF, we found that mothers with a previous history of neonatal death and current SSC uptake strongly predicted EBF at discharge and D28. At discharge, however, having a history of previous preterm birth or neonatal death increased the odds of EBF while multiple current births, current VLBW delivery, and the use of standard binders decreased the likelihood of EBF.

### Predictors of KMC

Our findings show that the implementation phases substantively increased the uptake of effective KMC at discharge and D28. Overall, the coverage of effective KMC at discharge and D28 was 70.6% and 67.1%, respectively. This increase in KMC uptake at discharge is comparable to those reported in other implementation research studies that adopted locally-specific KMC scale-up phases [13,25]. Our study of all three phase periods demonstrated that KMC uptake at D28 was similar to discharge. Our uptake of 71% at discharge approaches the Ethiopian Ministry of Health’s national 80% KMC target. Our finding of facilitators and barriers to effective KMC may provide clinicians with factors to more effectively target KMC support while admitted and at home after discharge through health extension or community-level interventions.

The predictors of effective KMC at discharge and D28 varied. Of note, the 3-fold increase in KMC uptake among mothers with a previous history of neonatal death may represent a compensatory mechanism, where a mother readily adopts KMC in order to prevent the death of their current newborn. For example, a mother who had once experienced the death of a child may be more likely to seek and adhere to the advice of health professionals (e.g. attending antenatal care (ANC) or providing KMC) compared to mothers who had never lost a previous newborn. This adoption of healthy practices may be a way of combating the negative emotional states often reported among mothers with preterm birth/LBW delivery, and a proactive attempt to improve parental self-image and responsibility [30,31]. On the other hand, we found that mothers with multiple births and those with assisted births through caesarean sections or forceps had significantly lower odds of practising KMC. This could be related to the high burden of child care experienced by such mothers and the difficulty of providing breastfeeding/KMC position to two or more newborns while recovering from a birth intervention such as a caesarean section. This finding is similar to those reported by Kymre et al. [32], where mothers with caesarean section were unable to practice SSC with their LBW infants for extended periods. One potential solution to this problem is to encourage fathers or close family members to initiate SSC for mothers with c-sections or to intermittently co-provide SSC to twins/multiples. Furthermore, we discovered that mothers with partners whose age was similar to their own (≤3-year gap) were more likely to practice KMC. Potentially, couples who are of the same age may be more likely to work in synergy and to share home responsibilities more equitably. However, such synergy may tend to depreciate as the age gap between partners widens. Women with older spouses may have more difficulties voicing their needs for instrumental support for KMC. For example, younger mothers may be able to obtain support with household chores from older male partners in a context where home/household care is traditionally considered to be a female’s responsibility [13,33].

### Predictors of SSC

The refinement of the implementation phases led to a significant increase in SSC uptake, especially at D28, with a 35% increase from phase 0 to 2. This increase in KMC uptake at D28 is very important, as it shows that the final implementation phase may have successfully mitigated the primary factors that account for large drop-offs in SSC after discharge. The only negative predictor of SSC was having multiple concurrent births; it is logistically more difficult to provide SSC for multiple newborns simultaneously. Having partners or family members provide SSC support may be a powerful strategy to increase SSC for this at-risk group. Concurrent breastfeeding, however, was a very strong positive predictor of SSC both at discharge and D28 and this finding is in line with that of several studies that show practicing SSC is associated with increases in exclusive breastfeeding – especially among LBW newborns [34,35].

### Predictors of EBF

Overall EBF was high in this study; approximately 75% of infants were exclusively breastfed both at discharge and D28. However, unlike our observation for KMC and SSC, there was a decrease in EBF from phase period 0 to 1 and 0 to 2. This decrease in EBF is unexpected; further evaluation may be needed to uncover why the higher rates of KMC and SSC in phases 1 and 2 did not translate into a concomitant increase in EBF rates in both phases. Intensive support for EBF among a few participants at fewer implementation sites during phase 0 may have resulted in higher EBF uptake in phase 0. This high initial EBF may have tapered off slightly as the number of facilities and mothers to support increased over the phase periods.

Our study detected several predictors that decrease the likelihood of EBF among mothers of LBW newborns. Global evidence shows that EBF and early initiation of breastfeeding can prevent 20% of deaths in the neonatal period [36]. Our study showed that EBF at discharge was two times higher among women with a history of neonatal death. Furthermore, multiple current births and very LBW delivery were significant negative predictors of EBF. These two factors are known to increase the likelihood of hospitalization and mother-infant separation in the neonatal intensive care unit, which may contribute to lowering breastfeeding initiation and exclusivity [22]. Interestingly, our results show that mothers who did not own mobile phones were 60% less likely to practice EBF and sufficient SSC 28 days after discharge. This may suggest mothers with more economic resources and/or social support through phone contact may be more likely to practice KMC.

### Relevance for practice and research

Results from this study identified factors that increase or decrease the likelihood of families practicing KMC, SSC, and EBF at discharge and through 28 days of life. In the Ethiopian context, these predictors can be incorporated into the design of effective KMC scale-up phases to help achieve the ambitious national coverage target of ≥80%. Furthermore, these findings can equip health extension workers or clinicians at health centres or primary/general/specialty hospitals with the knowledge of which specific low birth weight newborn, maternal/family, and sociodemographic factors decrease the likelihood of adopting effective KMC practices. For example, those with multiple births and/or very LBW newborns may require additional coaching and support to initiate and sustain KMC. Furthermore, only 40% of pregnant mothers in this study achieved the WHO recommendation of receiving four or more ANC visits during pregnancy. Since ANC or post-natal care may provide unique opportunities to counsel mothers on the importance of KMC and share coping strategies for practicing KMC in difficult contexts (e.g. with multiple births).

### Limitations

We created models to predict KMC, SSC, and EBF using multivariate logistic regression with a backward stepwise approach to identify relevant and significant predictors of SSC, EBF, and KMC. While this method is commonly used in statistical modelling, it does have its limitations. These may include the risk of overfitting the model, variable selection bias, and the decision of what variable to include is primarily p-value driven. Since this was an implementation study, there may be selection bias in the recruited mothers who delivered at one of five hospitals in Amhara, Ethiopia. Thus, these findings may not fully represent predictors of KMC among home-birthed newborns. Further, KMC, SSC, and EBF practices at D28 were self-reported and may be subject to social desirability bias. Future studies may benefit from a wider range of facility types, country/regions, and potential predictors, as well as use more *a priori*-defined data collection methods to increase rigor and reproducibility.

## CONCLUSIONS

There are increasing global efforts to increase the quality and coverage of KMC to promote survival among LBW newborns [11]. However, we identified that the predictors of KMC and its two components – SSC and EBF – are not universal. Rather, we identified divergent factors predicting KMC and its two components. Yet, KMC, EBF, and SSC do share several predictors both at discharge and D28 although there is variability in predictors for these three practices at different time points. Consequently, families/mothers with multiple births warrant additional, targeted KMC support. Not surprisingly in a KMC program emphasizing SSC and EBF, practicing SSC was the largest positive predictor of practicing EBF and EBF was the largest predictor of SSC. Another key driver in KMC and SSC uptake was the increasing refinement of the KMC implementation phases over time and the hospital at which KMC was delivered. Collectively, the divergence of KMC uptake by hospitals at D28 (range of aORs = 3.0–4.6) and by model period (range of aOR = 3.2–5.0) suggests context-specific and facility-specific factors play a dramatic role in KMC uptake. Hospitals and health systems implementing KMC for the first time will likely benefit from cycles of improvement and optimisation like those used in the parent study [13]. Using the protective and supportive factors we identified for KMC and its two components – EBF and SSC – may provide KMC clinicians and government KMC planners more evidence to effectively target KMC, EBF, and SSC support. This approach to targeted KMC support may be critical for advancing KMC coverage among vulnerable newborns globally and supporting Ethiopia’s 80% KMC target by 2025.

## References

[1] Cutland CL Lackritz EM Mallett-Moore T Bardají A Chandrasekaran R Lahariya C Low birth weight: Case definition & guidelines for data collection, analysis, and presentation of maternal immunization safety data. Vaccine. 2017;35:6492–500. 10.1016/j.vaccine.2017.01.049 29150054 PMC5710991

[2] Hack M Klein NK Taylor HG Long-term developmental outcomes of low birth weight infants. Future Child. 1995;5:176–96. 10.2307/1602514 7543353

[3] McCormick MC The contribution of low birth weight to infant mortality and childhood morbidity. N Engl J Med. 1985;312:82–90. 10.1056/NEJM198501103120204 3880598

[4] Upadhyay RP Naik G Choudhary TS Chowdhury R Taneja S Bhandari N Cognitive and motor outcomes in children born low birth weight: a systematic review and meta-analysis of studies from South Asia. BMC Pediatr. 2019;19:35. 10.1186/s12887-019-1408-8 30696415 PMC6350290

[5] Barker DJ The fetal and infant origins of adult disease. BMJ. 1990;301:1111. 10.1136/bmj.301.6761.1111 2252919 PMC1664286

[6] World Health Organization. Recommendations for care of the preterm or low-birth-weight infant. Geneva: World Health Organization; 2022. Available: https://www.who.int/publications/i/item/9789240058262. Accessed: 13 June 2024.

[7] Masaba BB Mmusi-Phetoe RM Neonatal Survival in Sub-Sahara: A Review of Kenya and South Africa. J Multidiscip Healthc. 2020;13:709–16. 10.2147/JMDH.S260058 32801733 PMC7398680

[8] Ethiopian Public Health Institute, International Classification of Functioning, Disability and Health. Ethiopia Mini Demographic and Health Survey 2019: Final report. Rockville, Maryland, USA: Ethiopian Public Health Institute, International Classification of Functioning, Disability and Health. Ethiopia Mini Demographic and Health Survey; 2021. Available: https://dhsprogram.com/pubs/pdf/FR363/FR363.pdf. Accessed: 13 June 2024.

[9] Girma D Dejene H Adugna L Predictors of Neonatal Mortality in Ethiopia: A Comprehensive Review of Follow-Up Studies. Int J Pediatr. 2022;1:1491912. 10.1155/2022/1491912 35189632 PMC8856832

[10] Seidman G Unnikrishnan S Kenny E Myslinski S Cairns-Smith S Mulligan B Barriers and enablers of kangaroo mother care practice: a systematic review. PLoS One. 2022;10:e0125643. 10.1371/journal.pone.0125643 25993306 PMC4439040

[11] Martines JC Portela A Bahl R Developing context-specific models to achieve high coverage and quality of KMC in India and Ethiopia: Learnings from implementation research. Acta Paediatr. 2023;112 Suppl 473:3–5. 10.1111/apa.16862 37519119

[12] World Health Organization. Kangaroo mother care: a practical guide. Geneva: World Health Organization; 2003. Available: https://www.who.int/publications/i/item/9241590351. Accessed: 13 June 2024.

[13] Gobezayehu AG Lijalem M Endalamaw LA Mohammad H Beyene T Mekonnen TB Creation of a globally informed and locally relevant KMC implementation model for population-impact in Amhara, Ethiopia. Acta Paediatr. 2023;112 Suppl 473:42–55. 10.1111/apa.16587 36544262

[14] Weldearegay HG Medhanyie AA Abrha MW Tadesse L Tekle E Yakob B Quality of Kangaroo Mother Care services in Ethiopia: Implications for policy and practice. PLoS One. 2019;14:e0225258. 10.1371/journal.pone.0225258 31756225 PMC6874352

[15] United Nations International Children’s Emergency Fund, World Health Organization. Ending preventable newborn deaths and stillbirths by 2030: Moving faster towards high-quality universal health coverage in 2020–2025. New York, USA: United Nations International Children's Emergency Fund; 2020. Available: https://www.unicef.org/reports/ending-preventable-newborn-deaths-stillbirths-quality-health-coverage-2020-2025. Accessed: 13 June 2024.

[16] Mony PK Tadele H Gobezayehu AG Chan GJ Kumar A Mazumder S Scaling up Kangaroo Mother Care in Ethiopia and India: a multi-site implementation research study. BMJ Glob Health. 2021;6:e005905. 10.1136/bmjgh-2021-005905 34518203 PMC8438727

[17] World Health Organization. Kangaroo mother care: a transformative innovation in health care. Geneva: World Health Organization; 2023. Available: https://www.who.int/publications-detail-redirect/9789240072657. Accessed: 13 June 2024.

[18] World Health Organization. Kangaroo mother care: implementation strategy for scale-up adaptable to different country contexts. Geneva: World Health Organization; 2023. Available: https://www.who.int/publications-detail-redirect/9789240071636. Accessed: 13 June 2024.

[19] Muhe LM McClure EM Nigussie AK Mekasha A Worku B Worku A Major causes of death in preterm infants in selected hospitals in Ethiopia (SIP): a prospective, cross-sectional, observational study. Lancet Glob Health. 2019;7:e1130–8. 10.1016/S2214-109X(19)30220-7 31303299 PMC6639243

[20] Conde-Agudelo A Díaz-Rossello JL Kangaroo mother care to reduce morbidity and mortality in low birthweight infants. Cochrane Database Syst Rev. 2016;2016:CD002771. 10.1002/14651858.CD002771.pub4 11034759

[21] Pérez-Escamilla R Tomori C Hernández-Cordero S Baker P Barros AJD Bégin F Breastfeeding: crucially important, but increasingly challenged in a market-driven world. Lancet. 2023;401:472–85. 10.1016/S0140-6736(22)01932-8 36764313

[22] Parker MG Stellwagen LM Noble L Kim JH Poindexter BB Puopolo KM Promoting Human Milk and Breastfeeding for the Very Low Birth Weight Infant. Pediatrics. 2021;148:e2021054272. 10.1542/peds.2021-054272 34635582

[23] Kinshella MW Hiwa T Pickerill K Vidler M Dube Q Goldfarb D Barriers and facilitators of facility-based kangaroo mother care in sub-Saharan Africa: a systematic review. BMC Pregnancy Childbirth. 2021;21:176. 10.1186/s12884-021-03646-3 33663415 PMC7934357

[24] Johnston C Campbell-Yeo M Disher T Benoit B Fernandes A Streiner D Skin-to-skin care for procedural pain in neonates. Cochrane Database Syst Rev. 2017;2:CD008435. 10.1002/14651858.CD008435.pub3 28205208 PMC6464258

[25] Estifanos AS Haile Mariam D Fikre A Kote M Tariku A Chan GJ Implementation science to design, test and scale up effective Kangaroo Mother Care in Oromia region, Ethiopia. Acta Paediatr. 2023;112 Suppl 473:56–64. 10.1111/apa.16413 35691617

[26] Beyene SA Hadush MY Gebregizabher FA Gebremariam DS Asmelash T Zelelow YB Achieving high coverage of Kangaroo mother care practice is possible: Lessons from implementation research for accelerating scale-up in Tigray Region, Ethiopia. Acta Paediatr. 2023;112 Suppl 473:77–85. 10.1111/apa.16374 35651289

[27] Medhanyie AA Alemu H Asefa A Beyene SA Gebregizabher FA Aziz K Kangaroo Mother Care implementation research to develop models for accelerating scale-up in India and Ethiopia: study protocol for an adequacy evaluation. BMJ Open. 2019;9:e025879. 10.1136/bmjopen-2018-025879 31753865 PMC6886988

[28] Garcia KKS Abrahão AA Research Development Using REDCap Software. Healthc Inform Res. 2021;27:341–9. 10.4258/hir.2021.27.4.341 34788915 PMC8654330

[29] Omary C Wright P Kumarasamy MA Franks N Esper G Mouzon HB Using Routinely Collected Electronic Health Record Data to Predict Readmission and Target Care Coordination. J Healthc Qual. 2022;44:11–22. 10.1097/JHQ.0000000000000318 34294659

[30] Trumello C Candelori C Cofini M Cimino S Cerniglia L Paciello M Mothers’ Depression, Anxiety, and Mental Representations After Preterm Birth: A Study During the Infant’s Hospitalization in a Neonatal Intensive Care Unit. Front Public Health. 2018;6:359. 10.3389/fpubh.2018.00359 30581812 PMC6293875

[31] Miles MS Holditch-Davis D Parenting the prematurely born child: pathways of influence. Semin Perinatol. 1997;21:254–66. 10.1016/S0146-0005(97)80067-5 9205979

[32] Kymre IG Bondas T Balancing preterm infants’ developmental needs with parents’ readiness for skin-to-skin care: a phenomenological study. Int J Qual Stud Health Well-being. 2013;8:21370. 10.3402/qhw.v8i0.21370 23849269 PMC3710397

[33] Asfaha MD Comeau DL Spangler SA Spratt BL Alamineh L Gobezayehu AG Neonatal care and community-level treatment seeking for possible severe bacterial infection (PSBI) in Amhara, Ethiopia. BMC Health Serv Res. 2020;20:264. 10.1186/s12913-020-05081-0 32228682 PMC7106804

[34] Wang Y Zhao T Zhang Y Li S Cong X Positive Effects of Kangaroo Mother Care on Long-Term Breastfeeding Rates, Growth, and Neurodevelopment in Preterm Infants. Breastfeed Med. 2021;16:282–91. 10.1089/bfm.2020.0358 33533688

[35] Heidarzadeh M Hosseini MB Ershadmanesh M Gholamitabar Tabari M Khazaee S The Effect of Kangaroo Mother Care (KMC) on Breast Feeding at the Time of NICU Discharge. Iran Red Crescent Med J. 2013;15:302–6. 10.5812/ircmj.2160 24083002 PMC3785903

[36] Jones G Steketee RW Black RE Bhutta ZA Morris SS Bellagio Child Survival Study Group How many child deaths can we prevent this year? Lancet. 2003;362:65–71. 10.1016/S0140-6736(03)13811-1 12853204

